# MAP kinase phosphatase-1 - a new player at the nexus between sarcopenia and metabolic disease

**DOI:** 10.18632/aging.100135

**Published:** 2010-04-06

**Authors:** Rachel J. Roth Flach, Anton M. Bennett

**Affiliations:** Department of Pharmacology and Program in Integrative Cell Signaling and Neurobiology of Metabolism, Yale University School of Medicine, New Haven, CT 06520, USA; ^1^ Current Address: Program in Molecular Medicine, University of Massachusetts Medical School, Worcester, MA 01605, USA

**Keywords:** MAP kinase phosphatase, MAP kinase, sarcopenia, metabolism

## Abstract

Sarcopenia, which is defined by the loss of skeletal muscle mass, predisposes skeletal muscle to metabolic dysfunction which can precipitate metabolic disease. Similarly, overnutrition, which is a major health problem in modern society, also causes metabolic dysfunction in skeletal muscle and predisposition to metabolic disease. It is now the prevailing view that both aging and overnutrition negatively impact skeletal muscle metabolic homeostasis through deleterious effects on the mitochondria. Accordingly, interplay between the molecular pathways implicated in aging and overnutrition that induce mitochondrial dysfunction are apparent. Recent work from our laboratory has uncovered the stress-responsive mitogen-activated protein kinase (MAPK) phosphatase-1 (MKP-1) as a new player in the regulation of metabolic homeostasis in skeletal muscle and mitochondrial dysfunction caused by overnutrition. These observations raise the intriguing possibility that MKP-1 may function as a common target in the convergence between sarcopenia and overnutrition in a pathophysiological pathway that leads to a loss of skeletal muscle mitochondrial function. With the increasing aging population it will become more important to understand how MKP-1, and possibly other phosphatases, operate at the nexus between sarcopenia and metabolic disease.

Skeletal muscle is a major contributor to resting levels of energy expenditure [1], and reduced skeletal muscle function contributes to age-induced obesity and diabetes. Disruption of skeletal muscle function may also contribute to insulin resistance and diabetes caused by overnutrition and obesity [2]. There is increasing evidence that similar molecular pathways are at work in promoting age-related and obesity-related muscle dysfunction. These pathways seem to converge at the level of mitochondrial dysfunction.

The overall metabolic capacity of skeletal muscle is determined by the myofiber type of the particular muscle. Though less abundant, oxidative/slow twitch (MHC type I, IIa) muscle fibers are important for overall metabolic wellness. However, the most abundant myofibers are glycolytic or fast twitch (MHC type IIx/IIB) muscle fibers which mainly utilize glycolysis to metabolize glucose. Oxidative myofibers have increased mitochondria, more blood flow, and are resistant to fatigue. In contrast, glycolytic fibers have the least amount of mitochondria, and fatigue easily [2,3].

Skeletal muscle fiber type establishment is regulated by changes in calcium flux, nervous system inputs, and transcriptional events and is a dynamic process. Endurance exercise increases oxidative capacity of skeletal muscle, and to this end, human studies have shown that endurance athletes have increased amounts of MHC type I [2]. During periods of inactivity, fibers switch from oxidative to more glycolytic types [4]. Furthermore, an increased proportion of glycolytic fibers have been demonstrated in the elderly population as well as diet-induced obese populations [2,3]. However, it is not well understood whether these changes in myofiber content are causal to age- and overnutrition-induced obesity and insulin resistance, or whether they are merely consequences of these events.

One hallmark of the aging process is the progressive atrophy of skeletal muscle leading to weakness and frailness, a condition termed sarcopenia, which is also associated with increased obesity in elderly [5]. It is thought that the occurrence of sarcopenia in the elderly is related to a switch in myofiber composition, a loss of muscle stem cell number, and decreased mitochondrial function [6,7]. To this end, mitochondrial number and function decreases with age in human subjects [8,9]. Numerous hypotheses have surfaced as to why mitochondrial function decreases with age. These hypotheses include increased reactive oxygen species (ROS) production, chronic inflammation and/or mitochondrial DNA damage [8,10-14]. One may draw many comparisons between the decline in skeletal muscle function that occurs with aging and mitochondrial dysfunction in the skeletal muscle of individuals that consume high levels of calories. Recent work has demonstrated that mitochondrial damage occurs in obesity due to enhanced ROS and chronic inflammation caused by increased fatty acid load [15-17]. These data suggest that modulation of fatty acid-driven signaling pathways, inflammatory pathways, or ROS may prevent mitochondrial damage and thus, enhance skeletal muscle oxidative capacity, therefore improving overall metabolic performance.

**Figure 1.**
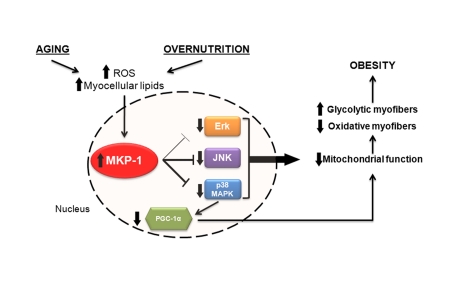
Potential relationship between aging, overnutrition and MKP-1-mediated regulation of skeletal muscle mitochondrial function.

## Molecular targets controlling skeletal muscle function and their impact on aging

*Calcineurin/NFAT* - Through transgenic and knockout mouse models, calcineurin, a calcium-activated phosphatase (also known as protein phosphatase 2B), has been shown to be necessary to drive the slow twitch MHC phenotype [18-20]. Calcineurin does this by controlling the phosphorylation status of a transcription factor called nuclear factor of activated T-cells (NFAT), which is activated upon dephosphorylation. Active NFAT enhances transcription of genes that promote a slow-twitch myofiber phenotype [18,21]. Interestingly, calcineurin has been demonstrated to increase in activity with age in a ROS-dependent manner, and correlates with age-induced muscle dysfunction [22].

*MAP Kinases*** -** The MAPKs consist of growth factor-regulated extracellular signal related kinases 1 and 2 (ERK1/2), and the stress-activated MAPKs, c-jun NH_2_-terminal kinase (JNK) and p38 MAPK [23]. MAPKs are activated by phosphorylation on regulatory tyrosine and threonine residues by upstream MAP kinase kinases (MKKs), and are inactivated by dephosphorylation on these same regulatory residues by MAPK phosphatases (MKPs) [23,24]. Although it is appreciated that MAPKs play an essential role in myogenesis, relatively little is known about the involvement of the MAPKs in fiber type establishment. A role for ERK1/2 has been demonstrated in slow-fiber type expression, as constitutively active Ras increases MHC type I expression [25]. There is also evidence that p38 MAPK is able to drive MHC type IIx (intermediate) gene expression in myoblasts [26].

Increased MAPK activity after exercise has been shown to be important for exercise-mediated gene expression, which may contribute to the role of exercise ameliorating the effects of aging in skeletal muscle [27]. Though the contribution of MAPKs in aging has not been convincingly addressed in vertebrates, some studies in *drosophila* as well as *c. elegans* have demonstrated a role for MAPKs for enhancement of lifespan. Whereas the role of p38 MAPK in promotion of lifespan is controversial [28,29], JNK activity enhances lifespan by antagonizing insulin signaling [30,31]. Additionally, decreased ERK1/2 activity throughout aging is known to promote senescence [32]. These data suggest the possibility that decreased MAPK activity during aging may have deleterious effects on metabolic health and lifespan.

*PGC-1α* - The transcriptional co-activator peroxisome proliferator-activated receptor gamma co-activator 1-α (PGC-1α) drives mitochondrial biogenesis and function and additionally is important in the cellular defense against reactive oxygen species [33,34]. Specifically, in skeletal muscle, the expression of PGC-1α drives not only mitochondrial biogenesis and oxidative myofiber establishment but also vascularization [35-37]. It has been found that a high fat diet or fatty acid treatment causes a reduction in the expression of PGC-1α and other mitochondrial genes in skeletal muscle [38-40], which may be a mechanism through which excess caloric intake impairs skeletal muscle function.

Recent work has also demonstrated that transgenic overexpression of PGC-1α in skeletal muscle improves sarcopenia and obesity associated with aging in mice [41]. This implies that pharmacological control of stability or expression of PGC-1α may be a therapeutic option for the elderly to ameliorate metabolic dysfunction. Pharmacological modulation of protein stability remains somewhat challenging and so alternative strategies targeting regulators of PGC-1α function are likely to meet with more success. Stability of PGC-1α has been shown to be driven by both acetylation as well as phosphorylation [42,43]. Stability and activity of PGC-1α is enhanced by phosphorylation by p38 MAPK [43] which again raises the interesting issue of the role of the MAPKs in both sarcopenia and in responses to overnutrition.

*MAP Kinase Phosphatases*** - **MKPs are dual-specificity phosphatases that constitute a sub-family of the protein tyrosine phosphatase (PTP) superfamily [44]. MKPs are classified into three subgroups based on subcellular localization, tissue distribution and substrate preference [44,45]. MKP-1 is the founding member of this family of enzymes and is a nuclear-localized, immediate-early gene that is responsive to numerous stimuli including ROS, cytokines, growth factors, and fatty acids [15,46]. MKP-1 expression is confined to the nucleus and therefore is restricted to the dephosphorylation of the nuclear pool of active MAPKs [47]. Though MKP-1 has the ability to dephosphorylate all three MAPKs, it displays substrate preference to the stress-responsive MAPKs, p38 MAPK and JNK [48,49].

As discussed, enhanced ROS, cytokines and fatty acids can induce the expression of MKP-1 [15,46]. Therefore, it is conceivable that increased MKP‑1 expression in response to stresses such as these might play a role in the demise of skeletal muscle mitochondrial function. Indeed, we had provided the first insight into a potential connection between mitochondrial function and MKP-1 [47]. We showed that in mice lacking MKP-1 mitochondrial oxidative phosphorylation was enhanced in the oxidative portions of skeletal muscle [47]. Consistent with this MKP-1-deficient mice exhibit enhanced levels of energy expenditure and were resistant to diet-induced obesity [47]. These results provide genetic evidence that MKP-1 is connected, through modulation of the MAPKs, to mitochondrial function in skeletal muscle as well as whole body energetics. It is tempting to speculate that age- and diet-induced stresses that promote deleterious mitochondrial function could be attributed to the convergence, at least in part, to the overexpression of MKP-1 in skeletal muscle.

More recently we have been able to provide mechanistic insight in to the pathway linking MKP-1 to mitochondrial function. We have found that MKP-1 is a key regulator of the master controller of mitochondrial biogenesis, PGC-1α [15]. When mice are fed a high-fat diet, the expression levels of MKP-1 in skeletal muscle increase which results in a reduction in PGC-1α levels. In contrast, PGC-1α levels are maintained in mice lacking MKP-1 [15]. Consistent with the notion that PGC-1α promotes oxidative myofiber composition MKP-1-deficient mice are refractory to the switch from oxidative to glycolytic myofibers seen in wild type mice fed a high fat diet and remain lean. These observations support the idea that MKP-1 plays an important role in maintaining skeletal muscle health. Mechanistically, MKP-1 appears to control a pool of nuclear p38 MAPK activity that is responsible for phosphorylating PGC-1α on residues that promote its stability. Therefore, increased MKP-1 expression levels result in a reduction of p38 MAPK-mediated PGC-1α phosphorylation and ultimately, stability. Collectively, these observations raise the possibility that impairment in mitochondrial function might arise through disturbing the dynamic homeostatic balance between MKP-1 and p38 MAPK activity in the nucleus. The precise contribution of MKP-1, as well as other MKPs which have the capacity to dephosphorylate p38 MAPK in the nucleus, to mitochondrial dysfunction in either aging or overnutrition still remains to be fully realized. Nevertheless, these recent insights provide a platform from which these questions can be addressed.

## Phosphatases in aging

The role of PTPs in the aging process is largely unknown. On a broad scale, enhanced phosphatase activity has been associated with cellular senescence [32,50]. It is also recognized that ROS, which inhibit PTPs by modification of the catalytic cysteine [51], are increased during the aging process. The enhanced levels of ROS during aging are likely due to the increased production of pro-inflammatory cytokines [13,52], and reduced antioxidant defenses [13]. However, even though ROS inhibit phosphatase activity, enhanced ROS levels are also known to drive cellular senescence [53], suggesting a potential role for PTPs both in promoting as well as inhibiting senescence. ROS also elicits a cellular stress response, which may induce stress-responsive phosphatases, such as the MKPs, to serve as negative regulators of excessive MAPK activity [54,55]. More studies are warranted to determine what role enhanced ROS in a whole organism has on modulation of PTP activity during aging.

In support of the data that similar mechanisms are at work in overnutrition as well as age-induced metabolic dysfunction, our laboratory has demonstrated that mice lacking MKP-1 are resistant to age-induced obesity, in the absence of a high fat diet [47]. Moreover, MKP-1 expression in skeletal muscle of aged human patients has been shown to be enhanced [56]. Though the mechanisms behind the resistance to age-induced obesity in mice lacking MKP-1 have not yet been defined, one may hypothesize that similar mechanisms also apply as those in a high fat diet scenario.  For example, increases in cellular insults such as ROS and intramyocellular lipids, which increase during aging [57,58], may trigger increased MKP-1expression, thus negatively regulating p38 MAPK activity, and therefore, PGC-1α stability. Consistent with this notion, as previously discussed, PGC-1α expression is sufficient to relieve rodents of age-related sarcopenia [41]. It is unclear however, how ROS may promote increased levels of MKP-1 expression without affecting catalytic activity. Nevertheless, modulation of p38 MAPK activity by MKP-1 may be important in the progression of age-related sarcopenia and obesity.

## Treatments for sarcopenia

*Caloric Restriction*** - **Calorie restriction is perhaps the most well recognized anti-aging therapy, and exerts this effect in multiple ways [13]. Calorie restriction has an anti-inflammatory effect and is important for decreasing oxidant production during aging [13]. In addition, calorie restriction is associated with decreased MAPK activity [13]. Provocatively, calorie restriction may serve to prevent the reported increase in MKP-1 levels during aging. Increased MKP-1 levels during aging would reduce PGC‑1α levels thereby removing the protective effects of stressors on skeletal muscle. Calorie restriction also activates the deacetylase SIRT1, which is a positive regulator of longevity in *c. elegans*[59]. To this end, SIRT1 also promotes PGC-1α activity by deacetylation [42], thus demonstrating a role for calorie restriction in promotion of PGC-1α activity. Interestingly, acetylation is also known to enhance the phosphatase function of MKP-1 [60]. One hypothesis may be that SIRT1 deacetylates, and therefore decreases activity of MKP‑1, leading to increased PGC-1α function, thus, preventing myofiber dysfunction.

*Exercise*** -** Moderate exercise retards the aging process, and additionally induces an oxidative muscle phenotype [13]. Several hypotheses have arisen regarding the role of exercise in aging. Leading hypotheses include the fact that a moderate exercise regimen reduces pro-inflammatory cytokine production and chronic inflammation. The reduced level of inflammation leads to a decrease in ROS production, which lessens DNA damage [13]. Other groups have demonstrated that resistance training enhances myofiber size and contractility, thus preventing age-mediated myofiber loss [61,62]. Importantly, exercise has been shown to induce MAPK activity in skeletal muscle, which is important for exercise-mediated gene expression [27]. In particular, p38 MAPK is known to drive PGC-1α transcription as well as stability in skeletal muscle [43,63]. In animals lacking MKP-1 expression, it would be important to determine if the enhanced MAPK activity in skeletal muscle promotes similar gene expression events as exercise. If this is the case, mice lacking MKP-1 may exhibit characteristics similar to that of the molecular effects of exercise.

## Perspective

It is now well accepted that aging is an underlying predisposition to metabolic disease. Not surprisingly, the pathways that succumb to the deleterious effects of aging often seem to be those that also control metabolism. Protein tyrosine phosphatases have not featured as prominent targets for convergence for these two pathophysiological processes up until now. MKP-1 appears to exhibit characteristics that afford it the ability to respond to age-related stresses such as increased ROS and free fatty acids in skeletal muscle. If in fact MKP-1 plays a major role in the convergence in skeletal muscle between aging and susceptibility to metabolic disease, how this phosphatase is regulated by such external factors needs to be addressed in significantly more depth. One intriguing, and as yet to be tested idea, is whether MKP-1 is more deeply intertwined with other pathways that control longevity such as the SIRTs. As such, it will be interesting to determine whether there is a longevity phenotype in mice lacking MKP-1. Clearly, these ideas set the stage for further work to be conducted on age- and nutrition-related consequences, not only on MKP-1, but also other protein tyrosine phosphatases in skeletal muscle.
